# Rapid postnatal suspicion of tracheal agenesis facilitated by point-of-care airway ultrasound: a case report

**DOI:** 10.1186/s40981-026-00870-8

**Published:** 2026-07-31

**Authors:** Akihisa Shindo, Hirotsugu Kanda, Tomoyuki Maruyama, Sarah Kyuragi Luthe, Tomoyuki Kawamata

**Affiliations:** Department of Anesthesiology, Wakayama Medical University School of Medicine, 811-1 Kimiidera, Wakayama City, 641-8509 Wakayama Pref. Japan

**Keywords:** Tracheal agenesis, Airway ultrasound, Point-of-Care Ultrasound(POCUS)

## Abstract

**Background:**

Tracheal agenesis is a rare congenital airway malformation that may cause unexpected cannot ventilate, cannot intubate (CVCI) events at birth. Early recognition is critical, but the role of point-of-care airway ultrasound remains unclear.

**Case presentation:**

A male neonate (35 + 1 weeks) was delivered by emergency cesarean section due to fetal distress, with prenatal findings suggesting duodenal atresia. Immediately after birth, he developed severe cyanosis and bradycardia. Bag-mask ventilation was ineffective, and intubation attempts failed despite glottic visualization. Point-of-care airway ultrasound demonstrated tracheal discontinuity, raising suspicion of tracheal agenesis. Esophageal intubation was performed, resulting in effective ventilation and stabilization. Tracheal agenesis (Floyd type II) was subsequently confirmed by computed tomography. The patient survives at 23 months with long-term ventilation via cervical esophagostomy.

**Conclusion:**

Point-of-care airway ultrasound may facilitate rapid suspicion of tracheal agenesis and guide life-saving airway management in neonatal CVCI.

**Supplementary Information:**

The online version contains supplementary material available at https://doi.org/10.1186/s40981-026-00870-8.

## Introduction

Tracheal agenesis is a rare congenital anomaly of uncertain etiology that can lead to unexpected cannot ventilate, cannot intubate (CVCI) events immediately after birth [1]. In many cases, a tracheoesophageal fistula is present, allowing partial ventilation via esophageal intubation [1–3]. Definitive diagnosis typically relies on imaging modalities such as computed tomography (CT), which demonstrate tracheal discontinuity. Delayed recognition can therefore be fatal, particularly because effective ventilation may require esophageal intubation, an approach that is not routinely performed in standard airway management.

A previous report has described a case definitively diagnosed using flexible bronchoscopy or CT [1, 4]. However, to the best of our knowledge, the use of point-of-care airway ultrasound to facilitate early recognition in this context has not been reported. Here, we describe a neonate delivered by emergency cesarean section due to fetal distress who developed CVCI immediately after birth and in whom tracheal agenesis was promptly suspected using point-of-care airway ultrasound. This case report was prepared with reference to the Anaesthesia Case Report (ACRE) checklist [5].

## Case report

The patient’s mother was a 34-year-old woman with no significant medical history. At 32 weeks and 0 days of gestation, prenatal ultrasonography revealed a double-bubble sign and polyhydramnios, suggestive of duodenal atresia; no other abnormalities were identified. An emergency cesarean section was planned at 35 weeks and 1 day of gestation due to fetal distress. Anesthesia was performed using combined spinal-epidural anesthesia.

At birth, the neonate did not initiate spontaneous respirations and exhibited profound generalized cyanosis with absent muscle tone. The heart rate was 80–90 beats/min. Supplemental oxygen administration and bag-mask ventilation were attempted; however, effective ventilation could not be achieved. At that time, oxygen saturation was unobtainable, and the heart rate had decreased to 40 beats/min. We therefore attempted endotracheal intubation. Although the glottis was clearly visualized using a laryngoscope, only the tip of a 3.5-mm inner-diameter tracheal tube could be advanced approximately 5 mm beyond the vocal cords, but effective ventilation could not be achieved. Further advancement was not possible because of marked resistance. Downsizing to a 3.0-mm inner-diameter tube also failed to facilitate passage, ventilation remained impossible, and mild swelling was observed in the lower cervical region. At that time, the heart rate was 40 beats/min and the SpO₂ was 40%.

**Fig. 1:**
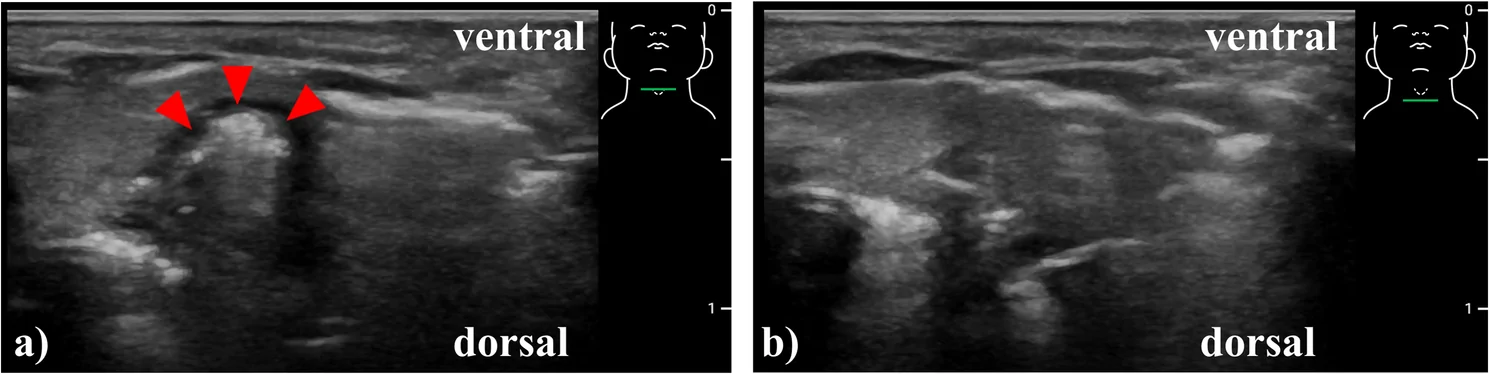
Point-of-care airway ultrasonography. **a** On ultrasonographic images obtained just below the glottis, the thyroid cartilage appears as a high-intensity structure, indicated by the red arrowheads. **b** As the ultrasound probe was moved approximately 1 cm caudally from just below the glottis, the thyroid cartilage, seen as a high-intensity structure, disappeared. The scale bars in both panels represent 1 cm, and the green lines in the schematic diagrams indicate the probe positions

We performed airway ultrasonography using a SonoSite PX® device to investigate the cause of failed tracheal tube insertion. On ultrasonographic images obtained just below the glottis, the thyroid cartilage was visualized as highly echogenic structures (Fig. 1a). As the probe was moved distally, the thyroid cartilages gradually disappeared, and the tracheal lumen was no longer visible (Fig. 1b). Based on these findings, we strongly suspected tracheal atresia. We therefore performed esophageal intubation with a 3.5-mm inner-diameter tube, resulting in effective ventilation, resolution of cyanosis, restoration of the heart rate to 110 beats/min, and an increase in SpO₂ to 80%. Apgar scores were 1 at 1 min, 1 at 5 min, 1 at 10 min, and 4 at 20 min. Please refer to Table 1 for the detailed chronological course. Anthropometric measurements showed a length of 44.5 cm and a weight of 1,774 g (− 1.84 SD, standard deviation). 

**Table 1:** Timeline of postnatal events

Clock time	Time after birth	Clinical events and interventions
18:50	0 min	The neonate was delivered by cesarean section in the operating room.
18:51	1 min	No spontaneous respiration. Generalized cyanosis and absent muscle tone were observed. Supplemental oxygen and bag-mask ventilation were initiated.
18:53	3 min	SpO₂ could not be measured. Heart rate was 80–90 beats/min.
18:55	5 min	Heart rate decreased to 40 beats/min.
18:59	9 min	Endotracheal intubation with a 3.5-mm inner-diameter endotracheal tube was attempted but was unsuccessful.
19:00	10 min	Intubation with a 3.0-mm inner-diameter endotracheal tube was also unsuccessful.
19:03	13 min	Point-of-care airway ultrasound was performed, raising suspicion of tracheal agenesis.
19:05	15 min	Esophageal intubation was performed using a 3.5-mm inner-diameter endotracheal tube, resulting in effective ventilation.
19:06	16 min	Heart rate recovered to 110 beats/min, and oxygen saturation increased to 80%.
19:26	36 min	Oxygen saturation increased to 98%.
19:29	39 min	The neonate was transferred from the operating room to the neonatal intensive care unit (NICU).

**Fig. 2:**
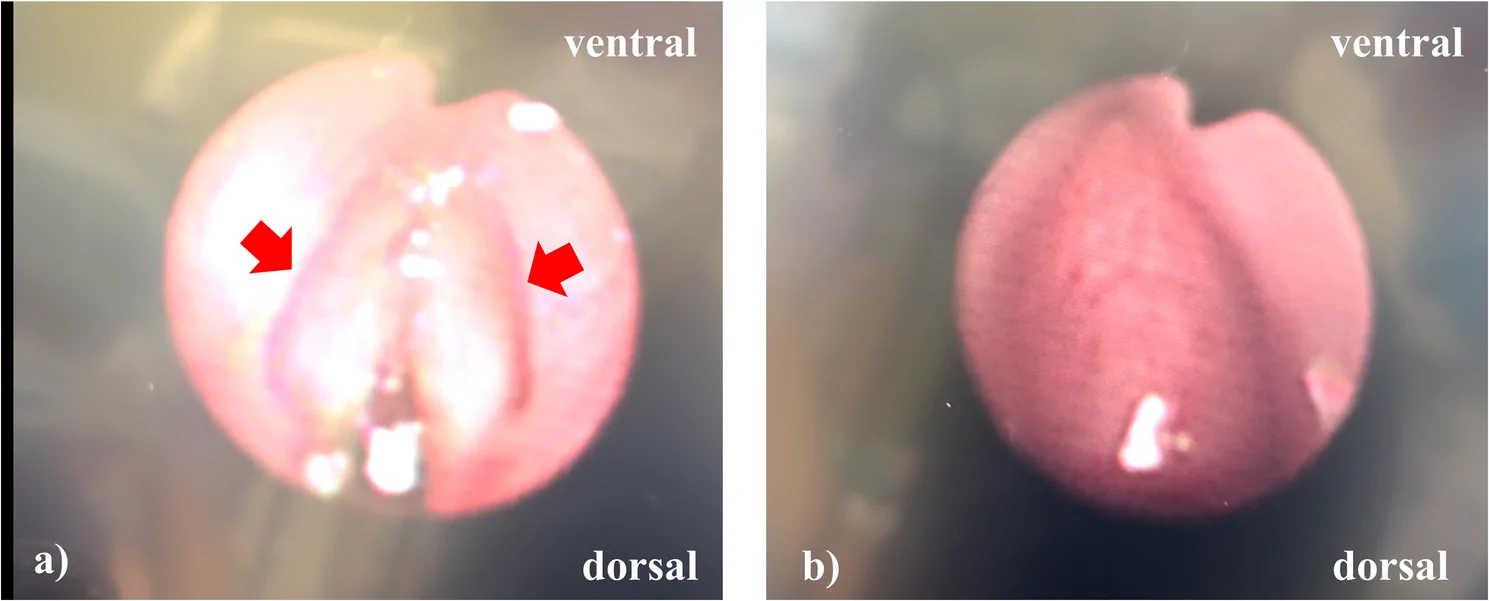
Laryngeal endoscopy. **a** The vocal cords are indicated by the red arrows. **b** An image obtained just below the vocal cords. The image shows abrupt termination of the airway and absence of the trachea at the expected location

**Fig. 3:**
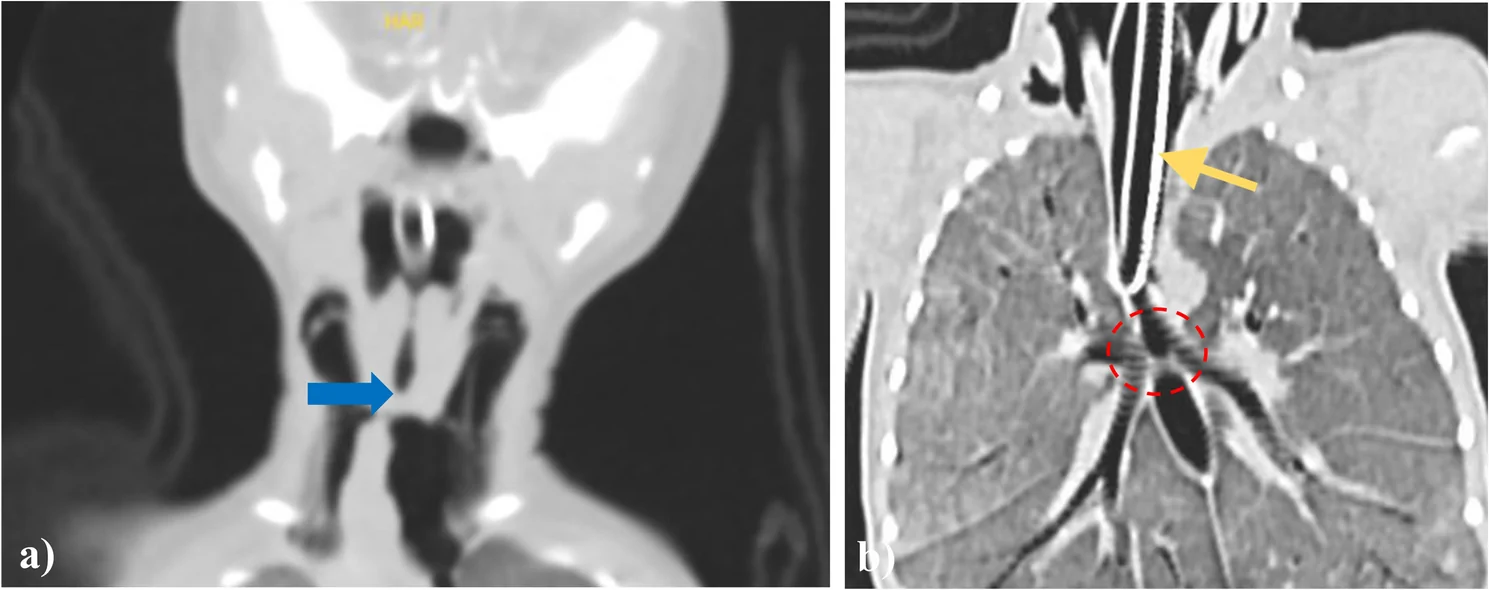
Computed tomography after resuscitation. **a** Coronal section of the neck showing the blind-ending trachea indicated by the blue arrow. **b **This is a coronal section of the chest. The yellow arrow indicates an endotracheal tube inserted into the upper esophagus, and the area outlined by the red dashed line demonstrates continuity between the esophagus and trachea

**Fig. 4:**
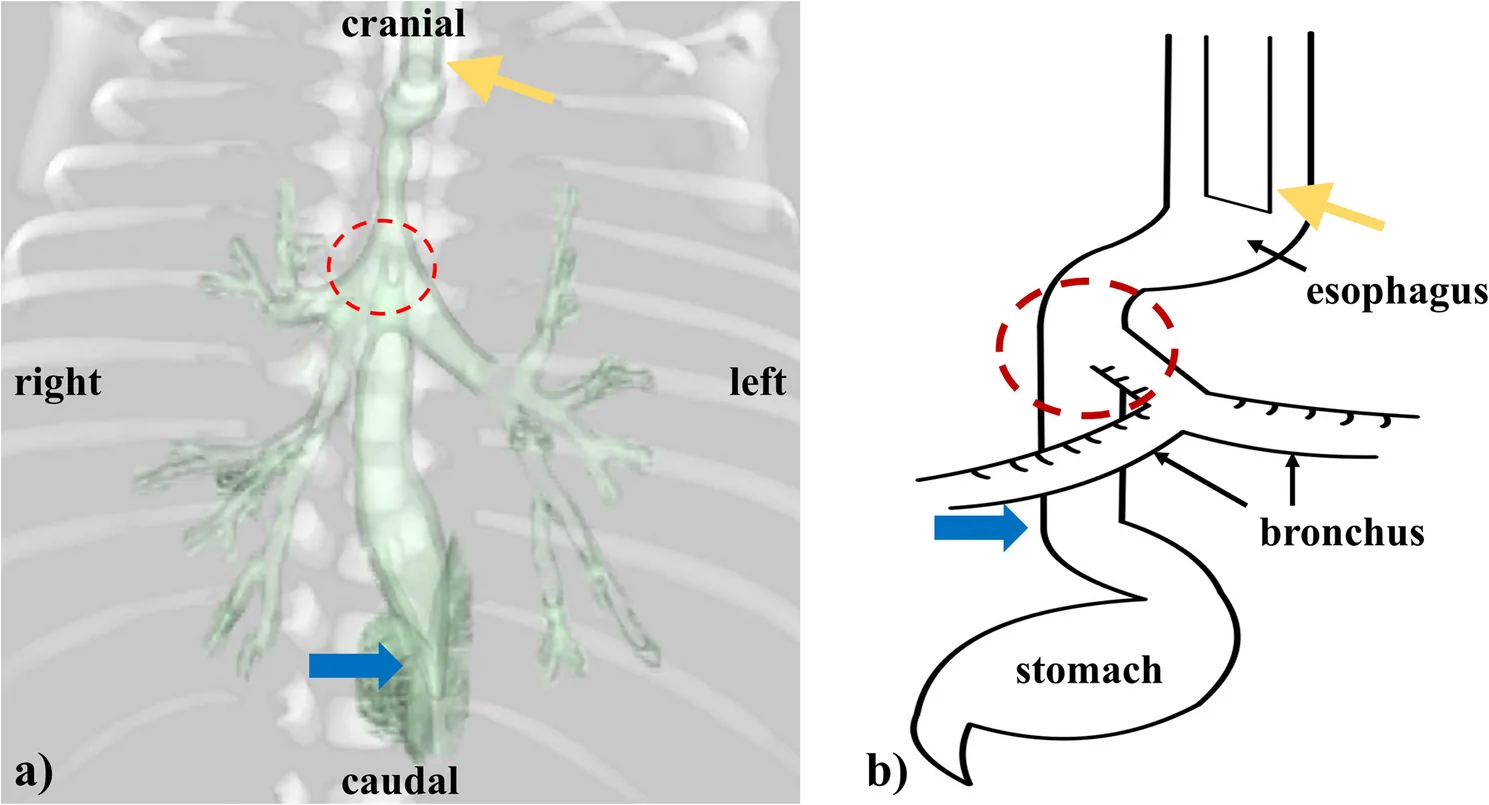
Three-dimensional computed tomography and schematic representation of tracheal agenesis. **a** The green-colored structures indicate the patient’s esophagus and trachea. The yellow arrow indicates an endotracheal tube inserted into the upper esophagus. The blue arrow indicates the site of gastroesophageal banding. The area outlined by the red dashed line represents a tracheoesophageal fistula, which communicates with the right and left main bronchi. **b **This schematic diagram was created on the basis of the 3D-CT images. The yellow arrow indicates an endotracheal tube inserted into the upper esophagus. The blue arrow indicates the site of gastroesophageal banding. The area outlined by the red dashed line represents a tracheoesophageal fistula. 3D-CT; three-dimensional computed tomography

Flexible bronchoscopy revealed complete occlusion of the trachea at the laryngeal level, approximately 5 mm distal to the glottis, preventing advancement of the endotracheal tube (Fig. 2). After stabilization of the patient’s general condition, CT was performed. Imaging demonstrated a blind-ending trachea (Fig. 3a). In addition, communication between the esophagus and trachea was identified, explaining why ventilation could be achieved via esophageal intubation (Fig. 3b). To prevent gastroesophageal reflux, aspiration, and gastric insufflation, esophageal banding and gastrostomy were performed on the day of birth. Postoperatively, CT was repeated, and three-dimensional CT images were reconstructed based on these findings (Fig. 4). The images showed that the entire the main bronchi joined at a normal carina, which communicated with the esophagus via a tracheoesophageal fistula. Based on CT findings, a definitive diagnosis of tracheal agenesis (Floyd type II) with concomitant duodenal atresia was established.

Definitive repair of the duodenal atresia was performed on postnatal day 3. An enterostomy was created on postnatal day 10, followed by construction of a cervical esophagostomy, functionally equivalent to a tracheostomy, on postnatal day 17. Written informed consent for publication was obtained from the patient’s parents.

## Discussion

Tracheal agenesis is a rare congenital anomaly defined by partial or complete absence of the trachea, with an etiology that remains uncertain. The reported incidence is less than 1 per 50,000 live births, with a male predominance of approximately 2:1. Polyhydramnios is frequently associated with this condition; however, prenatal diagnosis remains challenging despite advances in fetal imaging [1, 3]. To the best of our knowledge, this is the first report describing the rapid postnatal suspicion of tracheal agenesis facilitated by point-of-care airway ultrasound.

Establishing a definitive diagnosis of tracheal agenesis in the neonatal period is particularly challenging when the condition presents as an unexpected CVCI event immediately after birth. Traditionally, flexible bronchoscopy and CT have been the principal modalities used to establish a definitive diagnosis. Flexible bronchoscopy allows direct visualization of the airway and reliable identification of the level of obstruction or discontinuity [6]. However, in critically unstable neonates, its use may be limited by the need for specialized equipment and operator expertise, as well as the potential to further compromise ventilation during the procedure. CT provides detailed anatomical delineation and enables accurate classification according to the Floyd classification system [7], which is essential for long-term management. Nevertheless, CT generally requires prior patient stabilization and transport to the imaging suite, which may be difficult during immediate postnatal resuscitation.

By contrast, airway ultrasound is a rapid, noninvasive bedside modality that can be performed without interrupting ongoing resuscitation. Previous studies have demonstrated its utility in evaluating pediatric airway anatomy, including management of difficult airway and confirmation of endotracheal tube placement [8–10]. In the present case, airway ultrasound enabled prompt identification of tracheal discontinuity after failed conventional intubation attempts, raising suspicion of tracheal agenesis before confirmation by flexible bronchoscopy and CT. This rapid bedside assessment was crucial in guiding the subsequent ventilation strategy and may have contributed to successful resuscitation. Although airway ultrasound cannot replace bronchoscopic or CT evaluation, it may facilitate early recognition of airway anomalies in emergent neonatal settings.

Tracheostomy may fail in cases of tracheal agenesis due to tracheal hypoplasia or distal airway discontinuity, and unsuccessful rescue cases have been reported [11]. When a tracheoesophageal fistula is present, esophageal intubation may provide effective ventilation [12]. Although detailed airway evaluation can be achieved by bronchoscopy and CT, there is often no time to perform these investigations in neonates presenting with a CVCI situation. When tracheal agenesis is suspected based on airway ultrasound findings, esophageal intubation, although typically avoided during neonatal resuscitation, should be considered as a life-saving intervention. Clinicians managing neonatal CVCI should therefore be aware that effective ventilation may be achieved by esophageal intubation in selected cases. In the present case, point-of-care airway ultrasound enabled rapid suspicion of tracheal agenesis, facilitating prompt selection of esophageal intubation and successful resuscitation.

In conclusion, point-of-care airway ultrasound enabled rapid postnatal suspicion of tracheal agenesis in this case. In neonates presenting with CVCI at birth, early application of airway ultrasound may provide critical information, allowing timely recognition of tracheal discontinuity and prompt selection of esophageal intubation as a life-saving airway strategy.

## Supplementary Information

Supplementary Material 1.

## Data Availability

Not applicable.

## Abbreviations

CVCI: Cannot ventilate, cannot intubate
CT: Computed tomography
