# Supplementary material for: Rapid postnatal suspicion of tracheal agenesis facilitated by point-of-care airway ultrasound: a case report
Source: JA Clin Rep. 2026 Jul 31;12:38. doi: 10.1186/s40981-026-00870-8 (PMC13719911; doi:10.1186/s40981-026-00870-8)
Supplement: Supplementary file 1 [file 40981_2026_870_MOESM1_ESM.pdf]

The narrative: A case report tells a story in a narrative format that includes the clinical context/challenges, assessment, pre-operative optimisation, anaesthetic interventions, critical incidents, outcomes and follow-up. The narrative should include a discussion of the rationale for any conclusions and any take-away messages.

Item name	Item no.	Brief description	Check
Title	1	Focused in terms of context, clinical challenges and intervention.	☑
Keywords	2	Two to five key words that identify topics in this case report.	☑
Summary	3	a) Why the case report is relevant to readers.	☑
		b) Case report:	☑
		- The procedure or situation for which peri-operative management was required.	☑
		- The principal clinical challenges.	☑
		- The strategies that were adopted to account for these challenges.	☑
		c) Conclusion - what the primary (and secondary) novel or educational message is.	☑
Introduction	4	a) A brief background to the clinical condition or problem presented.	☑
		b) Summarise why this case is unique and/or educational with medical literature references.	☑
Focused patient and contextual information	5	a) De-identified patient information.	☑
		b) The procedure or situation for which peri-operative management was required.	☐
		c) The main concerns of the patient.	☑
		d) Medical, family and psychosocial history.	☑
		e) Previous anaesthetics/interventions and their outcomes.	☑
		f) Physical examination and other clinical findings.	☑
Assessment, optimisation and consent	6	a) Assessment methods (e.g. laboratory/physiological testing, imaging, risk stratification).	☑
		b) Principal clinical challenges identified, e.g. comorbidities, incidents/events, clinical situation.	☑
		c) Optimisation interventions (e.g. pharmacological, physical, physiological).	☑
		d) Planned management (aims of management and strategies to address challenges).	☑
		e) Explanation of the consent process.	☑
Interventions *(specify device and manufacturer for all non-standard equipment)*	7	a) Monitoring.	☑
		b) Induction of anaesthesia/performance of regional anaesthesia, including:	☑
		- Pharmacology: drugs; doses; administration (include description of imaging/nerve localisation for regional anaesthesia); endpoints.	☑
		- Airway management if applicable.	☑
		c) Maintenance of anaesthesia/intra-operative management, including:	☑
		- Assessment of depth of anaesthesia/neuromuscular blockade function.	☑
		- Summary of physiological/physical observations.	☑
		- Summary of any interventions.	☑
		d) Emergence, including the early post-operative status of the patient.	☑
Adverse/critical events *(if applicable)*	8	a) Description of what was observed/detected and at what time/juncture.	☑
		b) Interventions to manage the event (e.g. pharmacological, airway, surgical, etc).	☑
		c) The success of interventions (including how this was assessed).	☑
		d) Human factors contributing to and/or mitigating the event (i.e. both negative and positive human factors).	☑
Follow-up and outcomes	9	a) Clinician- and patient-assessed outcomes, when appropriate.	☑
		b) Intervention success and tolerability (how was this assessed?)	☑
Discussion	10	a) Strengths and limitations in your approach to this case.	☑
		b) Discussion of the relevant medical literature.	☑
		c) The rationale for your conclusions.	☑
		d) The primary (and secondary) novel or educational message.	☑
		e) Suggested changes to future practice and/or research.	☑
Patient/carer perspective	11	Patients and/or carers should share their perspectives or experience whenever possible, including, when appropriate, information that would be useful in explanation to others undergoing similar peri-operative care.	☑
Acknowledgements	12	a) Statement as follows: "This case report was published with the written consent of the patient" (or next of kin, if applicable).	☑
		b) Declaration of any external funding and/or competing interests.	☑

©2021 Association of Anaesthetists
